# Age-Related Effect of Sleepiness on Driving Performance: A Systematic-Review

**DOI:** 10.3390/brainsci11081090

**Published:** 2021-08-19

**Authors:** Serena Scarpelli, Valentina Alfonsi, Maurizio Gorgoni, Milena Camaioni, Anna Maria Giannini, Luigi De Gennaro

**Affiliations:** 1 Department of Psychology, Sapienza University of Rome, Via dei Marsi 78, 00185 Rome, Italy; valentina.alfonsi@uniroma1.it (V.A.); maurizio.gorgoni@uniroma1.it (M.G.); milena.camaioni@uniroma1.it (M.C.); annamaria.giannini@uniroma1.it (A.M.G.); luigi.degennaro@uniroma1.it (L.D.G.); 2 IRCCS Fondazione Santa Lucia, Via Ardeatina 306/354, 00179 Rome, Italy

**Keywords:** sleepiness, drowsiness, driving, aging, older adults, EEG

## Abstract

Background: Several studies highlighted that sleepiness affects driving abilities. In particular, road traffic injuries due to excessive daytime sleepiness are about 10–20%. Considering that aging is related to substantial sleep changes and the number of older adults with driving license is increasing, the current review aims to summarize recent studies on this issue. Further, we intend to provide insights for future research. Methods: From the 717 records screened, ten articles were selected and systematically reviewed. Results: Among the selected articles, (a) five studies investigated sleepiness only by self-reported standardized measures; (b) two studies assessed sleepiness also using a behavioral task; (c) three studies obtained objective measures by electroencephalographic recordings. Conclusions: The available literature on the topic reports several limitations. Overall, many findings converge in evidencing that older drivers are less vulnerable to sleep loss and sleepiness-related driving impairments than young adults. These discrepancies in sleepiness vulnerability between age groups may be ascribed to differences in subjects’ lifestyles. Moreover, it has been hypothesized that older adults self-regulate their driving and avoid specific dangerous situations. We believe that an easy protocol to objectively evaluate the vigilance level in elderly and young adults is required, and further studies are needed.

## 1. Introduction

Driving is a crucial aspect of human functioning to keep a high quality of life and independence. The safety at the wheel is affected by multiple variables, such as visual abilities [1], attention and decision-making [2], age [3], and drowsiness [4]. Specifically, sleepiness has been recognized as one of the main factors that affect driving skills [5]. Sleepiness has been defined as difficulty maintaining wakefulness without external stimuli [6]. Excessive daytime sleepiness (EDS) is present in 10–20% of the general population and negatively impacts reaction time, vigilance, and judgment of performance at the wheel [5]. The sleepiness level can be evaluated by subjective or objective methods and may be influenced by poor sleep hygiene, circadian rhythm, or alcohol and drugs [7,8].

Epidemiological observations revealed that young and adult drivers (aged 15–44 years) account for 48% of road traffic deaths worldwide [9], and road traffic injuries due to sleepiness are about 10–20% [10,11,12]. Recent trends indicate that motor vehicle incidents are among the top five causes of mortality by 2030 [9]. For this reason, in the last decades, the relationship between driving performance and sleepiness received growing attention [5,13]. The available literature showed that fatal road accidents provoked by a driver falling asleep at the wheel are more common among young individuals [10,14,15,16,17]. Nevertheless, almost 7000 older adults were involved in motor vehicle accidents in the USA, considering that it has been estimated that more than 40 million elderly drivers have a driving license [18]. Although the absolute number of crashes involving older drivers is currently low, we have to note that the percentage of older people with a driving license has grown by 34% in the last decade [18], and crash frequency per mile driven begins to increase at around 65 years of age [19]. Despite accounting for only 8% of miles driven per year, older drivers appear to be involved in 14% of crash fatalities [19]. In particular, Italian data [20] showed that the drivers more frequently involved in road accidents were those aged 20–29 years (18.3%) and 40–54 years (29.2%), with a growing percentage (9.2%) of elders (≥70 years). This issue may be related to a rise in bodily damages and liability claims for property damage, especially among 75 years or older subjects [21].

Prior studies ascribed reductions in geriatric driving skills to cognitive, visual, and/or physical impairments [2,19,22,23,24,25]. However, sleepiness and sleep-related complaints—which strongly affect the other functions—have been rarely investigated. Actually, sleep patterns undergo significant changes during aging [26]: advanced bedtime and wake up time, decreased total sleep duration, fragmented sleep, and a substantial reduction of the slow-wave sleep, consistently with the significant lowering of slow-wave activity (SWA) during NREM sleep in the quantitative EEG [27,28]. Further, evidence highlighted older adults reported high rates of sleep disturbances, and approximately 20–30% of elderly people experience EDS [27]. Some authors hypothesized that older adults try to self-regulate their driving [2]. In other words, they avoid driving in high-risk settings, e.g., peak traffic times and dark conditions (e.g., [28,29]), which may contribute to reducing the consequences of their EDS and poor sleep quality.

It should be noted that by 2050 the proportion of the world’s population over 60 years will nearly double [30], and a better understanding of the complex relationship between sleepiness/vigilance degree, aging, and driving performance is becoming increasingly important.

This systematic review aims (a) to summarize and critically discuss recent studies on the relationship between sleepiness, aging, and driving skills; and (b) to provide insights and strategies to future research on this issue.

## 2. Materials and Methods

The systematic review included studies by following the PRISMA guidelines [31]. The study focused on published original quantitative studies on the relationship between sleepiness/drowsiness and driving performance in older adults. A search was conducted on PubMed, entering the following search terms in English: “Sleepiness” OR “Drowsiness” OR “Vigilance” AND “Driving” OR “Driving skills” AND “Older adults” OR “Elderly” OR “Aging”. Search fields were title, abstract, and keywords. Only quantitative research and original papers published in the last twenty years were further analyzed. Two experts selected eligible articles through a multi-step procedure (title reading, abstract, and full-text assessment). The literature search was completed with a manual search, reviewing the references contained in the selected articles.

**Figure 1:**
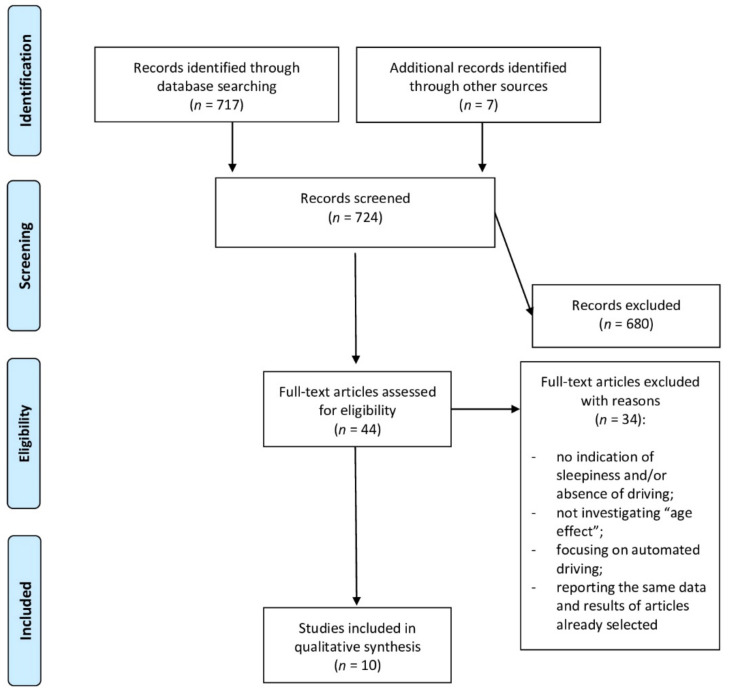
Flowchart of the article selection process.

Predefined inclusion criteria were: (1) papers including older adults (≥55 years) with data analysis, which explicitly considered the age factor or different age groups; (2) no subjects with relevant neurological problems and neurodegenerative diseases; (3) no studies exclusively aimed to assess the medications effect on sleepiness and/or driving; (4) protocols with an assessment of sleepiness by objective, subjective or behavioral methods; (5) protocols including driving performance evaluation through real driving, self-reported driving-related information, driving simulation or testing driving-related skills (no automated driving); (6) peer-reviewed articles (not just abstracts or conference papers); (7) no reviews, or meta-analyses; (8) articles written in English; (9) a year of publication between 2001 and 2021 (last twenty years). Duplicates were removed. Then, title and abstract screening was carried out. All potentially relevant papers were independently assessed for their eligibility. Studies that included older adults but not focusing on the difference between age groups were also excluded. A consensus session solved any disagreement between reviewers with a third reviewer. Figure 1 shows the flow chart of the article selection.

## 3. Results

Ten articles fulfilled the inclusion criteria [32,33,34,35,36,37,38,39,40,41]. Among the selected papers, (a) five studies investigated sleepiness only by self-reported standardized measures [34,35,36,37,40]; (b) two studies assessed sleepiness also using a behavioral task [39,41]; and (c) three studies obtained objective measures by electroencephalographic (EEG) recordings [32,33,38]. Table 1 summarizes the main characteristics and results of the ten papers included in the final study synthesis.

### 3.1. Self-Reported Sleepiness

The project by Vaz Fragoso and colleagues produced three studies on longitudinal data collecting from a sample of 430 older adults (≥70 years) [34,35,36]. They aimed to assess the relationship between drowsiness/sleep complaints and driving capacity. Both sleepiness and driving measures were self-reported in all studies [34,35,36]. Moreover, other sleep questionnaires to evaluate insomnia and sleep apnea risk were administered. Older adults had chronic disturbances, e.g., diabetes and hypertension, while a small part of the sample reported sleep apnea. As assessed by the Epworth sleepiness scale (ESS) [42], subjective sleepiness was related both to sleep apnea risk and chronic disease. At the baseline study [34], the authors revealed that subjects reporting low nighttime driving ability had significant insomnia symptoms with difficulties falling asleep or maintaining sleep, distress with sleep, and sleep-related impairment in functioning. Lower levels of driver self-ratings capacity and driving mileage were also associated with daytime sleepiness. Surprisingly, high scores in sleep apnea were not linked to driving measures, and sleep disturbances were not related to prior adverse driving episodes [34].

In a second study, the authors assessed the longitudinal association between sleep disturbances and adverse driving events in the same cohort of older adults [35]. Longitudinal evaluations of these episodes (i.e., crash or traffic infraction and near-crash or getting lost) were planned every six months for two years. Overall, 418 older drivers participated in the follow-up. Among the participants, 215 older drivers had at least one crash, traffic infraction, near-crash, or getting lost. Crashes appeared to be correlated with a traffic infraction and near-crash with getting lost. Subjects with these adverse events showed at the baseline higher ESS scores and greater driving frequency than older adults not experiencing these events at the wheel. However, it should be noted that the median ESS score was not clinically relevant [35].

Moreover, sleep disturbances did not significantly affect the odds of having adverse driving events [35]. In a third article, Vaz Fragoso and colleagues evaluated the relationship between sleep disturbances at the baseline and the subsequent driving cessation over a two-years period [36]. Results revealed that insomnia, daytime sleepiness, and sleep apnea risk were not longitudinally associated with driving cessation. Nevertheless, insomnia was the only sleep-related variable predicting the reduction of driving mileage. Moreover, multiple pharmacological treatments and age were associated with decreased mileage, while being male predicted increasing in daily driving mileage.

Another group aimed to cross-sectionally assess the relationship between near-crash/misses or accidents and sleepiness [37]. As in the previous studies, the ESS was used to evaluate self-perceived daytime sleepiness. Moreover, information about other disorders and driving behavior were collected from a sample of 4774 French drivers. Subjects were subdivided into four age groups: 18–30, 31–50, 51–65, and >65. Among the near misses, 46% were sleep-related. Interestingly, the regression model showed that being male, being young (18–30 years), being sensitive to caffeine, and having at least an episode of sleepiness at the wheel in the last year were the best predictors of near misses [37].

More recently, to compare the driving performance of younger and older drivers, Song et al. [40] recruited 68 healthy and cognitively intact subjects (29 younger vs. 39 older adults). They were tested through a driving simulator in two different conditions: (a) during monotonous driving simulation; and (b) during two 10-min driving sessions accompanied by an alertness-maintaining task (AMT). AMT aimed to promote vigilance in drivers and contained trivia questions on different topics. Finally, a visual analogue of fatigue scale was used to assess subjective levels of tiredness and stress. The authors found that AMT benefitted the younger group during the monotonous driving as compared with AMT condition. Younger drivers reported higher tiredness than older adults during the 50 min monotonous driving.

Moreover, AMT significantly advantaged the younger group who made more driving errors than older drivers during the first and second monotonous segments. Older adults did not show increased mistakes with fatigue. Surprisingly, AMT did not negatively impact their driving performance. However, they showed increased speed variability when driving with AMT [40].

### 3.2. Behavioral Task

Leufkens et al. [39] and Bartolacci et al. [41] used the psychomotor vigilance task (PVT) [43] as an instrument to assess psychomotor vigilance. The PVT is based on a simple visual reaction time test and is generally accompanied by a self-reported sleepiness rating (e.g., Karolinska sleepiness scale—KSS [44]). The study by Leufkens et al. [38] involved 63 subjects with 50–75 years who were subdivided into three groups: insomnia group with medications, insomnia group without medications, and healthy subjects. All participants performed the highway driving test [45], measuring tracking road performance. The standard deviation of lateral position (SDLP) was used as an index of individual driving performance. Moreover, other tasks were administered to assess driving-related skills (e.g., selective attention, decision making, stimulus interpretation, speed, and adaptive motor response to driving events; divided attention). A preliminary sleep assessment with polysomnography was performed, not finding differences between groups. Subjective sleepiness and sleep complaints were also assessed. Older good sleepers and older insomniacs did not differ in driving performance and driving-related skills, as well as in PVT performance. Reaction times at the PVT differed only between morning and evening performance [39]. Indeed, mean reaction time in the PVT was faster in the morning than the evening in controls, but not in subjects with insomnia.

More recently, Bartolacci et al. [41] compared 40 healthy older adults with 40 young subjects. Along with the subjective assessment of sleep quality and self-reported sleepiness, the PVT was administered to collect a behavioral sleepiness evaluation. Moreover, driving-related skills were tested: selective attention, tachistoscopic perception (e.g., the ability to obtain an overview, the skills about visual orientation, and the perceptual speed), and the risk assumption. Older adults reported lower sleep efficiency and worse performance in PVT (tendency to make more mistakes and slowing reaction times in the 10% of fastest responses) than the younger group. However, self-reported sleepiness was more significant in young subjects. In addition, older adults had lower performance in attention and tachistoscopic perception tests but appeared to be more cautious in traffic situations. The regression model showed that age was the only best predictor of cognitive driving-related skills, while sleepiness did not show any relation with these abilities. Excluding the age factor, the regression revealed that subjective sleepiness, PVT scores, and sleep quality tend to predict driving-related performances [41].

### 3.3. Electrophysiological Pattern

Campagne et al. [32], Lowden et al. [33], and Filtness et al. [38] assessed the relationship between sleepiness and driving by recordings EEG during a driving simulation. Specific features of the EEG recordings are described in Table 2. EEG recordings allowed the authors to consider alpha and/or theta power as indices of sleepiness.

Campagne et al. [32] compared older adults (60–70 years) with two other age groups (20–30 years and 40–50 years). One group was required to drive on a lighted motorway, while the other group drove on a nonlighted motorway condition. EEG was acquired at baseline periods before the driving test and during the monotonous and pro-longed night-driving simulation. No difference between the two lighting conditions and among the three age groups was found concerning the EEG patterns. Driving errors did not vary between the two lighting conditions. However, driving errors such as “running-off the road accidents” were more frequent in young subjects. Differently, speed variability was higher in older drivers, consistently with the observations of Song et al. [40]. Only young adults showed in both conditions a positive correlation between alpha power (high alpha power = low vigilance) and driving errors (i.e., running-off the road episodes on the left-hand side). Separate analyses on the two lighting conditions revealed a positive correlation between sleepiness and driving errors in the younger group during the lighted condition. For the older drivers, no correlation was found between any type of running-off the road errors and vigilance level assessed through the alpha power, whatever the lighted conditions. Differently, a positive correlation between the theta power and driving errors was found for older adults. Moreover, the theta power—representing a high level of sleepiness—was correlated to the speed variability in this group during a lighted condition. In the older group, excessive, low, and overall speed values were associated with the total number of running-off the road accidents.

Moreover, the analysis of the time course of the EEG power in the alpha and theta bands showed a significant increase in both indices during the prolonged driving task at night. Driving errors increased progressively as the number of laps increased.

A physiological assessment through EEG during the driving simulation was carried out also by Lowden et al. [33]. Ten young drivers (18–24 years) were compared with ten older drivers (aged 55–64 years). In this study, each subject participated in two 45-min driving simulations: (a) morning driving and (b) evening driving. The driving period was divided into 5-min bins (9 intervals), and the factor “time” was included in the analysis. Results showed that EEG power activity increased across the nine 5-min bins. In particular, alpha and sigma power (8–14 Hz) showed an increase. A main effect of age was observed for higher frequency band (12–32 Hz) that being increased during both conditions in older drivers. Age differences became bigger at the end of the night. Indeed, older drivers showed increased power in the frequency 10–16 Hz. Moreover, the sigma 1 band (12–14 Hz) increased across the time and was higher in older adults.

Further, after driving, increased salivary cortisol in older adults was found compared with the younger group. Subjective sleepiness evaluated by KSS was higher during the night driving in both groups. However, sleepiness appeared to be more pronounced in young subjects than older drivers during the night and at the end of the driving simulation.

The last EEG study by Filtness et al. [38] evaluated sleepiness at the wheel only in the early morning, considering two different situations: (a) after a regular night; and (b) after a sleep restriction. Twenty young subjects were compared with 19 older drivers. EEG was recorded during the driving task, and subjective sleepiness was assessed at regular intervals. After normal sleep, no difference between groups was found. Following sleep restriction, both groups had more driving accidents. A time effect was also observed: both age groups increased the number of incidents during the task. Moreover, in this condition, the younger group showed significantly more sleepiness-related incidents during driving simulation. Partly according to Campagne et al. [32], alpha and theta (4–11 Hz) EEG power was higher in younger drivers than older drivers. Consistently, subjective sleepiness (KSS) positively correlated with EEG measures after sleep restriction in both groups.

## 4. Discussion

A critical discussion of the selected articles may encourage the reflection on potential instruments and protocols to systematically assess the effect of sleepiness on driving performance in older adults.

Firstly, the current literature highlighted that the relationship between sleepiness and driving performance during aging is not linear. Self-reported studies by Vaz Fragoso et al. [34] revealed that daytime sleepiness and insomnia were related to lower self-perceived driving abilities and lower mileage, especially concerning nighttime driving. However, higher sleepiness at the baseline and a higher driving frequency were longitudinally associated with the probability of having a crash over the subsequent period, while clinically relevant sleep disorders were not associated with accidents [35]. These results suggest that both insomnia symptoms and sleepiness deserve attention in evaluating driving performance. This evidence is strengthened by the fact that higher insomnia scores and apnea risk at the baseline predicted a reduction in driving mileage at the follow-up [36]. To some extent, this is consistent with the hypothesis that older adults self-regulate their driving behaviors [2,46,47]. In other words, older drivers having sleep disturbances such as insomnia and/or EDS symptoms may change their driving practices avoiding high-risk settings or driving for shorter distances [36].

Moreover, it should be hypothesized that the relationship between driving, aging, and sleepiness is mediated by other factors, such as the quality of health and medical conditions [48]. Indeed, older drivers in the investigations by Vaz Fragoso et al. [34,35,36] were affected by mixed chronic pathologies such as diabetes, hypertension, and arthritis, despite the median scores of both tests assessing insomnia and EDS were in the normal range. It is worth noting that driving, especially during the night, required different intact functions (i.e., vision, attention, cognition) that in older adults may be impaired, and sleepiness could worsen the chronic conditions or be a consequence of these [49].

In this vein, pharmacotherapy may express a high medical burden that would influence both sleepiness and driving abilities [36,50,51,52]. On the one hand, it has been shown that the use of multiple medications longitudinally impacted the driving mileage [36]. On the other hand, it seems that older chronic hypnotics users did not show impaired driving behavior than healthy older drivers [39]. Actually, Leufkens et al. [39], comparing insomniacs and good sleepers, found differences only in the reaction time task (PVT). The fact that controls reported faster reaction time in the morning than evening was in line with the evidence that older adults tend to have a morning chronotype [53,54].

As previously mentioned, aging is associated with a phase advance of sleep-wake rhythm that would provoke more drowsiness during the evening than morning [26]. Consistently, some studies highlighted older adults avoided driving in the evening or at night [2]. In this respect, it should be noted that Bartolacci et al. [41] placed the experimental session in the afternoon between 4.00 and 7.00 p.m., so the time of the day may have influenced the outcomes in older drivers [55,56]. Indeed, the older adults’ performances were worse than the young group for the tachistoscopic perception test and selective attention test [41]. Nevertheless, Bartolacci and colleagues [41] did not reveal a significant correlation between driving-related skills and vigilance. Differently, in line with previous literature [57,58], younger drivers showed a high propensity to risk-taking (i.e., greater impulsiveness) than older adults [41], and being young represented a better predictor for traffic crashes [37]. This evidence seems to be—once again—partly in line with the idea that during aging, people tend to assume protective behaviors and self-regulate their driving performance because of the reduced cognitive driving-related abilities, such as selective attention and tachistoscopic perception [41].

Regarding the comparisons between sleepiness levels in different age groups, several studies found that younger adults reported higher subjective sleepiness during driving performances [33,40,41]. Consistently, younger subjects showed significant advantages at driving using a task aimed to promote vigilance (AMT) throughout monotonous drive [40]. Interestingly, older adults benefitted from AMT, not showing impaired sustained attention during the driving. Younger drivers could be more sensitive to sleepiness, as highlighted by previous studies [59]. Actually, several investigations reported that low vigilance in young drivers was related to road accidents [16,60].

The greater sensitivity to sleepiness at the wheel of young drivers appears to be supported by EEG investigations. For instance, Lowden et al. [33] observed that the older group had higher sigma power and greater cortisol levels than the young group. Both these indices would protect older adults from driving sleepiness-related errors. In other words, young drivers could more rapidly develop severe sleepiness during pro-longed driving, also affecting the odd of having crashes. Accordingly, Campagne and colleagues [32] found that younger subjects had more running-off-the-road incidents.

Further, alpha power, as an expression of objective sleepiness [61], was higher in the younger group during the lighted motorway condition. However, this investigation showed increased theta activity during driving in older adults, that may reflect a local sleep phenomenon [62,63]. Indeed, in previous studies on young samples, both alpha and theta power show specific patterns during the wake-sleep transition [61]. The theta activity exhibits a fronto-central maximum peak before and after sleep onset. At the same time, the alpha power shows occipital maximum peak before sleep onset and fronto-central maximum expression during post-sleep onset (e.g., [64,65]). The increase of theta power along with driving errors among elderly people would indicate that they made mistakes at the wheel only in severe drowsiness conditions able to provoke sleep attacks. Alternatively, since young adults showed driving errors already with lower sleepiness levels (i.e., related to alpha activity), the result could support—once again—the idea of a greater impact of low vigilance in younger as compared to older adults [32]. In this vein, Filtness et al. [38] demonstrated that, albeit both age groups had more driving accidents after partial sleep deprivation, younger participants were more impaired by this condition characterized by severe sleepiness. Indeed, after sleep restriction, the young group exhibited more sleepiness-related accidents during early morning driving, associated with the presence of alpha and theta power [38].

Overall, most of these findings indicate that older drivers are less vulnerable to sleep loss and sleepiness-related driving impairments than young adults [32,33,38,40,66]. These discrepancies in sleepiness vulnerability between age groups may be due to differences in subjects’ lifestyles. For instance, young participants could have poor sleep habits and a sort of “chronic sleep deprivation”, as hypothesized by previous literature [67,68], and observed in studies on the consequences of COVID-19 on sleep-wake rhythm [69,70,71].

It is worth noting that this conclusion should be taken with caution since some studies revealed that older adults might underestimate their level of sleepiness in the assessments by self-report instruments [72,73], and the time of the day in which the studies have been performed could have affected the results [38,41].

## 5. Limitations and Future Perspectives

The literature specifically aimed to better understand the relationship between sleepiness, aging, and driving behavior is sparse and presents several limitations.

Firstly, investigations on a large sample are scarce [34,35,36,37], and none of these have studied sleepiness and driving abilities providing a comparison between young and older adults. The existing longitudinal results highlighted that sleep complaints (i.e., insomnia, EDS) might influence the possibility to keep driving. Nevertheless, the main limitation of these studies was the adoption of protocols using exclusively self-reported instruments to evaluate both driving performance and sleepiness [34,35,36,37]. In this respect, we believe that prospective protocols may help shed light on the longitudinal effects of sleep quality and sleepiness on driving during aging by integrating objective methods to study drive abilities (i.e., driving simulator). Moreover, the longitudinal repeated measures would allow researchers to understand whether the observed changes of driving behavior in the geriatric population are ascribed to sleep loss/sleep complaints (i.e., state-like factors) or due to stable individual differences (i.e., trait-like factors).

Another issue to consider in the current literature is the composition of the samples. For example, some studies involved mainly males [32,34,35,36,38], making the results not representative of the driving population. In fact, many studies point to the fact that males are more willing to take risks than females in terms of risk behavior in road traffic than females [74,75]. Moreover, gender differences during aging should be assessed since females tend to have more sleep disturbances such as insomnia in the post-menopausal period [76,77].

In addition, the age ranges considered in the reviewed studies were not homogeneous. Some studies included in the same age group both participants with 55 and ≥65 years considering all “older adults” [38,39,40,41]. Future studies should take into account that the elderly population is defined a people aged 65 and over [78]. Moreover, distinct age ranges should be assessed to detect slight differences during the geriatric age: (a) youngest-old ranging 65–74 years; (b) middle-old ranging 75–84 years and (c) oldest-old ≥85 years [79].

It is worth noting that the older participants selected in the reviewed studies were mainly considered “healthy” [32,33,37,38,40,41] to guarantee a certain homogeneity between subjects. However, comorbidities [34,35,36,39,48] and drugs usage [48] are frequent during aging. Indeed, medical disorders can affect sleepiness level and, consequently driving abilities [48]. The Federal Motor Carrier Safety Administration (FMCSA) has reported a comprehensive review of the risk of car incidents for a variety of medical disorders [80,81,82,83,84]. The time course, severity state and treatment of the disease modulate the impact on daily performances [48]. Diabetes, chronic insomnia, restless leg syndrome, obstructive sleep apnea (OSA), and dementia with related medications can all contribute to sleep deprivation and/or EDS [85]. Several pharmacotherapies could alter vigilance, e.g., anticonvulsants, antihistamines, antidepressants, antipsychotics and narcotics, dopaminergic therapy, and benzodiazepines [48]. In particular, OSA has been recognized as a significant risk factor for motor vehicle crashes and affects approximately 10–20% of adults [48,83]. Nevertheless, it is frequently undiagnosed in the general population [86] and very few studies were directly aimed to investigate the relationship between OSA, aging, and driving abilities (e.g., [87]). Therefore, future investigations should consider this issue providing further evidence about risk and protective factors affecting driving performances in the context of medical disorders among elderly people.

The further methodological question concerns the identification of instruments to evaluate sleepiness. Although the EEG studies provide a reliable measure of sleepiness assessing specific cortical oscillations (e.g., alpha and theta power) [32,33,38], EEG does not represent an easy and quick tool to use. It should be mentioned that in Europe, the sleepiness assessment recently became mandatory for high-risk subjects who apply to renew their driving license. The European Union (EU) directive on driving licenses was made subject to mandatory implementation by all member state from 31 December 2015 [88]. Specifically, this directive was based on the recommendations from a working group established by the Transport and Mobility Directorate of the European Commission in 2012 [89]. Accordingly, the Italian Government has issued a decree stating the obligation to assess sleepiness in people suffering from narcolepsy, sleep apnea, and diseases EDS-related (i.e., Decreto Legge del Ministero delle Infrastrutture e dei Trasporti del 22 dicembre 2015). This situation required an easy protocol to objectively evaluate the vigilance level both in elderly and young adults. For instance, an attempt in this direction was provided by the European Obstructive Sleep Apnea Screening (EUROSAS) questionnaire for drivers, developed by the European Union Driver License Committee as a screening tool for OSA [89,90].

In this vein, the assessment of sleepiness through a short and easy-to-administer behavioral task, such as PVT lasting few minutes [43,91], should be considered. Further, the behavioral task, administered at different times of the day, should be associated with a driving simulation test. However, very little data are available on the relationship between PVT, driving, and aging [39,41] since the issue deserves further investigation.

## Figures and Tables

**Table 1:** Characteristics of the selected studies and main findings.

Authors and Date of Publication	Participants	Age	Sleepiness Measures	Driving Measures	Study Design	Main Findings
Campagne et al., 2004	46 healthy drivers (all males)	20–30 years (*n* = 21) 40–50 years (*n* = 13) 60–70 years (*n* = 16)	Objective measures: EEG recording during driving performance	Driving simulator: monotonous and prolonged night-driving situation Two conditions: (1) lighted motorway (2) non-lighted motorway	Between-group comparisons (conditions): (1) 25 subjects drove on a lighted motorway vs. (2) 21 subjects drove on a non-lighted motorway Correlational analyses	The young group had higher running-off- the road accidents than older adults. The older group had higher speed variability than younger adults. Alpha power and driving errors in lighted condition showed a positive correlation for younger subjects. Theta power and driving errors were positively correlated in older adults. Theta power was correlated also to the speed variability in the older group during a lighted condition.
Vaz Fragoso et al., 2008	430 drivers without cognitive or vision impairment (365 males)	≥70 years	Subjective measure: ESS Other sleep measures:ISI SACS	Self-reported driving performance (VAS): (1) Driving mileage (2) Adverse driving events	Cross-sectional/Epidemiological survey (Descriptive analyses only)	Older drivers with insomnia symptoms and daytime drowsiness reported lower levels of self-rated driving abilities, especially regarding nighttime driving.
Lowden et al., 2009	30 healthy drivers (15 males)	18–24 years (*n* = 20) 55–63 years (*n* = 10)	Subjective measure: KSS every 5-min Objective measure: EEG recording during driving performance; saliva cortisol collected before and after driving	Dynamic Hi-Fi driving simulator 45-min driving in: (1) Evening condition (2) Morning condition	Repeated measures: Condition: (1) Evening condition vs. (2) Morning condition	Subjective sleepiness increased across each drive and was higher among young drivers at night. Relative EEG power increased among older drivers for frequencies of 10–16 Hz. The sigma 1 frequency band (12–14 Hz) increase during the task in older drivers. Salivary cortisol levels after night driving were higher in older drivers than youngers subjects.
Sagaspe et al., 2010	4774 healthy drivers (45.7% males)	18–31 years (12.7%) 31–50 years (43.8%) 51–65 years (27.7%) ≥65 years (15.8%)	Subjective measure: ESS Other sleep measures:Self-reported sleep disturbances	Self-reported driving performance: (1) Accidents (2) Near-misses	Cross-sectional/Epidemiological survey	The best predictors of the near misses were: being male, being young (18-30 years), being sensitive to caffeine, and having at least an episode of sleepiness at wheel in the last year.
Vaz Fragoso et al., 2010	430 drivers without cognitive or vision impairment (365 men)	≥70 years	Subjective measure: ESS Other sleep measures:ISI SACS	Self-reported driving performance: (1) Driving mileage (2) Adverse driving events evaluated every 6 months for 2 years.	Longitudinal design	215 older drivers had at least one adverse driving events. Subjects with adverse driving events showed at the baseline higher ESS scores and higher driving frequency than older adults not experiencing adverse events.
Filtness et al., 2012	40 healthy drivers (all males)	20–26 year (*n* = 20) 52–74 years (*n* = 20)	Subjective measures: ESS, KSS Objective measure: EEG recording during driving performance	Driving simulator in the early morning Two conditions: (1) After a normal night (2) After sleep deprivation (5 h.)	Between-group comparisons: Younger vs. Older in both conditions: (1) After normal night (2) After sleep deprivation	After sleep restriction both groups had more driving accidents and increased the number of incidents during the task. After sleep restriction the younger group showed significantly more sleepiness-related incidents during driving simulation. Alpha and theta (4–11 Hz) EEG power was higher in younger drivers than older drivers. KSS scores were positively correlated with EEG measures after sleep restriction in both groups.
Vaz Fragoso et al., 2013	430 drivers without cognitive or vision impairment (365 males)	≥70 years	Subjective measure: ESS Other sleep measures:ISI SACS	Self-reported driving performance: (1) Driving mileage (2) Adverse driving events (3) Driving cessation	Longitudinal design	Insomnia, daytime sleepiness, and sleep apnea risk were not longitudinally associated with driving cessation. Insomnia at baseline predicted the reduction of driving mileage. Polypharmacy and age were associated with decreased mileage, while being male predicted increasing in daily driving mileage.
Leufkens et al., 2014	63 drivers (34 males): 21 insomniac with pharmacological treatment 21 insomniacs without medications 21 good sleepers	50–75 years	Subjective measure: KSS Behavioral measure: PVT Other sleep measures: preliminary PSG assessment; GSQS	Road tracking performance: (1) Morning session (2) Evening session	Between-group comparisons (three groups): Treated insomniacs vs. untreated insomniacs vs. good sleepers	Older good sleepers and older insomniacs did not differ in driving performance and driving-related skills, as well as in PVT performance. Good sleepers showed faster mean reaction times at the PVT in the morning than evening.
Song et al., 2017	68 healthy drivers (31 males)	18–30 years (*n* = 29) 54–88 years (*n* = 39)	Subjective measures: VAS to indicate tiredness rating	Driving simulator: (a) 50-min monotonous driving (b) 10-min monotonous driving as baseline (c) 10-min trivia game (AMT1) during driving (d) 10-min monotonous driving as baseline (e) 10-min trivia game (AMT2) during driving	Between-group comparisons: Younger vs. Older	Younger drivers reported higher tiredness than older adults during the 50-min monotonous driving. AMT significantly advantaged the younger group who made more driving errors than older drivers during the first and second monotonous segments. Older adults did not show increased errors with fatigue. AMT did not negatively impact on their driving performance, but they showed increased speed variability when driving with AMT.
Bartolacci et al., 2020	80 healthy drivers (45 males)	20–35 years (*n*= 40) 58–80 years (*n* = 40)	Subjective measure: ESS, KSS Behavioral measure: PVT Other sleep-related measures: PSQI	Driving-related skills Vienna system traffic: (1) Selective attention—COG (2) Tachistoscopic vision—ATAVT (3) Risk perception —WRBTV	Between-group comparisons: Younger vs. Older	Older drivers showed lower sleep efficiency and lower performance in PVT than younger drivers.Self-reported sleepiness was greater in young subjects. Older adults had lower performance in attention and tachistoscopic perception tests. Older adults were more cautious in traffic situations than the younger group. Age was the only best predictors of cognitive driving-related abilities.

EEG, electroencephalography; ESS; Epworth sleepiness scale; ISI; insomnia severity index; KSS, Karolinska sleepiness scale; SACS, sleep apnea clinical score; VAS, visual analog scale; PVT, psychomotor vigilance task; PSG, polysomnography; GSQS, Groningen sleep quality scale; AMT, alertness maintaining task; PSQI, Pittsburgh sleep quality index; COG, Cognitrone (Test-set DRIVESTA); ATAVT, adaptive tachistoscopic traffic perception test; WRBTV, Vienna risk-taking test traffic.

**Table 2:** Characteristics of electroencephalographic protocols.

Authors	EEG Measures	Recordings	Quantitative Analysis
Campagne et al., 2004	4 EEG channels: F3-A2, C3-A2, P3-A2, O1-A2	(1) Baseline periods before driving: eyes open—eyes closed (5 min) (2) During driving simulation	Theta (4–8 Hz) Alpha (8–12 Hz) Beta (12–25 Hz) (alpha + theta)/beta ratio
Lowden et al., 2009	3 EEG channels: Fz-A1, Cz-A2, Oz-Pz EOG EMG	During driving simulation	Theta (4–8 Hz) Alpha 1 (8–10 Hz) Alpha 2 (10–12 Hz) Sigma 1 (12–14 Hz) Sigma 2 (14–16 Hz) Beta 1 (16–24 Hz) Beta 2 (24–32 Hz) Total power (4–32 Hz)
Filtness et al., 2012	2 EEG channels: C3-A1, C4-A2 EOG EMG	During driving simulation	Theta (4–7 Hz) Alpha (8–11 Hz)

EEG, electroencephalography; EOG, electroculography; EMG, electromyography.
